# Rate of Postoperative Urinary Retention after Anterior Compartment Prolapse Surgery: A Randomized Controlled Trial Comparing Early versus Conventional Transurethral Catheter Removal

**DOI:** 10.3390/jcm12103436

**Published:** 2023-05-12

**Authors:** Nareenun Chansriniyom, Apisith Saraluck, Athasit Kijmanawat, Rujira Wattanayingcharoenchai, Komkrit Aimjirakul, Jittima Manonai Bartlett, Orawee Chinthakanan

**Affiliations:** 1 Department of Obstetrics and Gynaecology, Faculty of Medicine Ramathibodi Hospital, Mahidol University, Bangkok 10400, Thailand; 2 Division of Female Pelvic Medicine and Reconstructive Surgery, Department of Obstetrics and Gynaecology, Faculty of Medicine Ramathibodi Hospital, Mahidol University, Bangkok 10400, Thailand

**Keywords:** anterior compartment prolapse surgery, pelvic organ prolapse, postoperative urinary retention, transurethral catheter, early catheter removal

## Abstract

Background: Pelvic organ prolapse is a common condition of pelvic floor dysfunction in women, especially in adult vaginally parous and elderly women. Because of its anatomy, the anterior compartment has a significant effect on urinary symptoms. Anterior colporrhaphy and colpocleisis are major anterior compartment prolapse-related surgeries. As we know, postoperative urinary retention (POUR) is one of the most common complications following pelvic floor surgery. To prevent this complication, indwelling bladder catheterization is routinely applied. In contrast, to minimize risk of infection and the patient’s discomfort, the catheter should be removed as soon as possible. However, there is a lack of clarity regarding the optimal timing for catheter removal. Therefore, the aim of this trial is to compare the rate of POUR after anterior prolapse surgery between early transurethral catheter removal (24 h postoperatively) and our standard practice (on postoperative day 3). Methods: We conducted a randomized controlled trial among patients undergoing anterior compartment prolapse surgery between 2020 and 2021 at a university hospital. Women were randomized into two groups. After removal, if the second void residual urine volume exceeded 150 mL, POUR was diagnosed, and intermittent catheterization was performed. The primary outcome was the POUR rate. The secondary outcomes included urinary tract infection, asymptomatic bacteriuria, time to ambulation, time to spontaneous voiding, length of hospitalization, and patient satisfaction. Analysis was performed according to the intention to treat principle. The calculated sample size was 68 patients (34 patients in each group) for a 95% confidence interval, 80% power, 5% probability of type I error, and 10% data loss. Discussion: This study demonstrated that early catheter removal was comparable in POUR rate to conventional treatment with shorter hospitalization among patients undergoing anterior compartment prolapse surgery. Additionally, we observed no re-hospitalization owing to POUR. Therefore, early transurethral catheter removal is preferable following anterior compartment prolapse-related surgery.

## 1. Introduction

The prevalence of pelvic organ prolapse (POP) ranges from 40 to 50%, with a 3 to 12% symptomatic rate and an increasing trend [1,2]. Rates of POP surgery are also increasing owing to the aging of the population [3]. POP is a common condition of pelvic floor dysfunction in women, especially in adult vaginally parous and elderly women. POP is evaluated using the pelvic organ prolapse quantification (POP-Q) system. There are three main compartments in POP: anterior, posterior, and apical compartments. An anterior compartment prolapse has a significant effect on urinary symptoms, especially causing voiding difficulty in advanced POP because of anatomical structures [4]. For patients with severe prolapse symptoms, surgery has been the treatment of choice. A vaginal operation for pelvic organ prolapse should be considered based on which compartment prolapses; however, anterior colporrhaphy (AC) and colpocleisis are two common vaginal operations in the treatment of prolapse that affect the anterior compartment and can result in any postoperative voiding symptoms [5].

Postoperative urinary retention (POUR) is a frequent consequence of gynecologic surgery, occurring in 2.5–43% of cases, especially with surgical correction of urinary incontinence and pelvic organ prolapse [6]. Postsurgical changes in edema, inflammation, and pain are important factors in POUR [7]. Furthermore, prolapse repair can lead to changes in the urethra–vesical junction that can affect voiding. Moreover, surgery in the vaginal and retropubic spaces is likely to cause disruption of the perforating nerve branches, leading to transient neuropathy, which can affect bladder sensation and micturition [8]. Thus, to prevent this complication, indwelling bladder catheterization is routinely used. However, prolonged bladder catheterization may increase the chance of developing a urinary tract infection (UTI) and may prevent ambulation, prolong hospitalization, and also adversely affect postoperative well-being [9,10]. While there is no universal definition for POUR, it is characterized by impaired bladder emptying with an elevation in residual urine volume. A stricter definition involves quantified voided volume and postvoid residual volume (PVR). Even with a quantified PVR, cutoff values vary in range. Generally, a PVR of less than 100–200 mL is considered acceptable [10,11,12]. There are several ways to assess PVR; the gold standard is measurement using catheterization. An alternative to catheterization is transvaginal ultrasonography [13] and a bladder scan. A non-invasive three-dimensional portable transabdominal ultrasound device is used to calculate bladder volume by imaging pockets of fluid. Its accuracy, specificity, and negative predictive value are 90%, 91.0%, and 93.1%, respectively [6]. Owing to the advantages of bladder scans, this technique is currently common for assessing PVR.

At Ramathibodi Hospital (Thailand), our practice is to remove the bladder catheter on postoperative day 3. A previous retrospective cohort study revealed that the rate of POUR after pelvic floor surgery was approximately 7% [14]. Results of a previous RCT showed that early transurethral catheter removal at 24 h postoperatively seemed to benefit patients undergoing vaginal prolapse surgery in terms of lower incidence of UTI and a shorter hospital stay [12]. Additionally, a recent systematic review of perioperative intervention in prolapse surgery found no conclusive evidence for optimal timing for catheter removal [15]. This study aims to determine a suitable period to remove the transurethral catheter by determining the rate of POUR after anterior compartment-related prolapse surgery in a trial comparing early catheter removal (24 h postoperatively) with the usual practice (three days postoperatively).

## 2. Materials and Methods

### 2.1. Study Design

We conducted a randomized controlled trial at the Urogynaecology Clinic, Female Pelvic Medicine and Reconstructive Surgery (FPMRS) division, Ramathibodi Hospital, Mahidol University, Bangkok, Thailand, a tertiary care center, between March 2020 and February 2021. The study protocol was approved by the Committee on Human Rights Related to Research Involving Human Subjects, Faculty of Medicine, Ramathibodi Hospital, Mahidol University (COA. MURA2020/357).

### 2.2. Objectives

The study aimed to determine the optimal time of catheterization following anterior prolapse surgery. The main outcome was to assess the rate of POUR after anterior compartment prolapse-related surgery (anterior colporrhaphy and colpocleisis), in a trial comparing early transurethral catheter removal (24 h postoperatively) with our conventional practice (on postoperative day 3).

### 2.3. Participating Hospitals and Interventions

Patients were recruited from the Urogynaecology Clinic of Ramathibodi Hospital. Women were eligible for enrollment if they were scheduled for anterior compartment prolapse-related surgery (anterior colporrhaphy and colpocleisis), with or without concomitant surgery, and agreed to participate in the study. Other inclusion criteria were no urinary incontinence or neurologic disorders, not a candidate for mid-urethral sling surgery, no preoperative urinary retention (PVR > 150 mL) [12], and no preoperative UTI. Women who refused to participate or had intraoperative bladder or ureteral injuries were excluded. Due to the author’s institute practice, we typically do not perform mesh surgery to correct pelvic organ prolapse; therefore, no mesh surgery was performed on any participant in this study.

**Figure 1:**
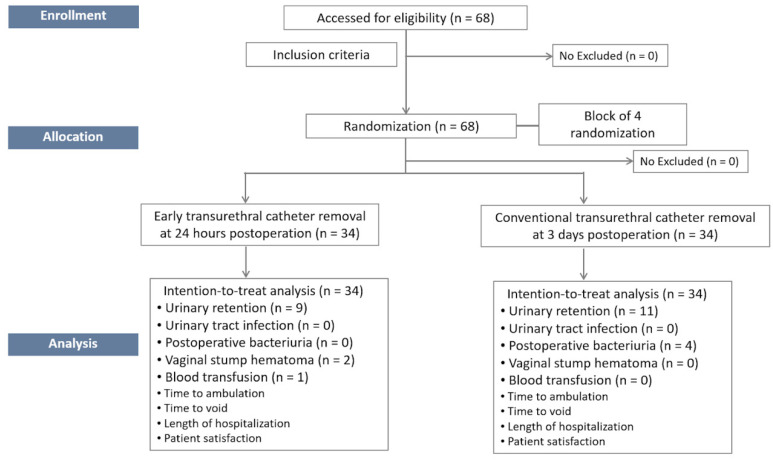
CONSORT 2010 flow diagram.

On the day of admission (the day before surgery), patients were personally informed about the risks and advantages of being enrolled prior to the randomization process. Demographic data and medical history were collected. POP-Q data were evaluated by a surgeon. Preoperative PVR was measured using transvaginal ultrasonography and calculated using the following formula: postvoid residual volume = (height × width × depth) × 0.7 [13]. Clinical symptoms and signs of UTI were recorded, and urinalysis with urine culture was performed in all patients to exclude UTI. Patients were randomly assigned to the early transurethral catheter removal (24 h postoperatively) or conventional removal (day 3 postoperatively) protocol on the day of surgery (Figure 1). Randomization was computer generated, with 1:1 group allocation in blocks of four. The randomization was concealed by a research assistant not involved in trial enrollment using consecutively numbered opaque envelopes, which were opened at the end of the procedures.

Preoperative antibiotic prophylaxis was administered to every patient within 60 min before the skin incision. Two grams of intravenous cefazolin was the antibiotic protocol of choice, unless the patient had drug allergies. Local infiltration of 0.5% xylocaine with adrenaline (1:200,000) was used to facilitate dissection. Then, either anterior colporrhaphy or colpocleisis was performed. In the anterior colporrhaphy procedure, a midline incision was made in the anterior vaginal wall, extending from the vaginal apex to the bladder neck but not beyond the urethra. The procedure was performed by a trained senior resident in obstetrics and gynecology, under the supervision of FPMRS staff, in all cases.

When the catheter was removed, micturition was recorded, and PVR was measured immediately using a bladder scanner (model BioCon-700, South Gloucestershire, UK). Postvoid bladder scanner standardized measurements were performed three times by two trained residents, with the average volume from six attempts recorded. Urine samples were sent for urinalysis and culture just before catheter removal. POUR was diagnosed with a second PVR exceeding 150 mL. Once POUR was detected, intermittent catheterization was performed. In case a fourth PVR still exceeded 150 mL, the patient was discharged, and the transurethral catheter was retained for a period of 3 days, followed by a visit to the outpatient department. The patients in both groups were scheduled for follow-up for 4 weeks after discharge. Before discharge, patient satisfaction was assessed using the Patient Global Impression Scale of Improvement (PGI-I) [16].

### 2.4. Outcomes

The primary outcome in this study was the rate of postoperative urinary retention in each group.

The secondary outcomes were the rates of postoperative UTI and asymptomatic bacteriuria (AB), mean time to ambulation, mean time to voiding, and mean length of hospital stay. The presence of AB was defined as a quantitative urine culture yielding at least 10^5^ colony-forming units of an identified single microorganism [17]. UTI was microbiological evidence of serious bacteriuria and pyuria, in addition to symptoms such as dysuria, increased bladder sensation, or fever [18]. The time to ambulation was defined as the interval between the completion of surgery and the time when the patient could stand up and walk, supported by a nurse or another person. The time to normal voiding was defined as the time interval between catheter removal and spontaneous voiding. The length of hospital stay was defined as the time interval between the completion of surgery and hospital discharge. Finally, PGI-I scores were used to assess patients’ overall satisfaction after prolapse surgery. There were seven possible responses (scored 1–7): (1) very much better, (2) much better, (3) a little better, (4) no change, (5) a little worse, (6) much worse, and (7) very much worse. The satisfaction level was defined as PGI-I 1 and 2.

### 2.5. Sample Size Calculation and Power Estimation

The sample size was derived from a previous study by Hakvoort et al. [11], using two independent proportions and a sample of 68 patients (34 patients in each group) for a 95% confidence interval (CI), 80% power, 5% probability of type I error, and 10% data loss.

### 2.6. Statistical Analysis

Stata/IC16 was used for statistical analysis (StataCorp LLC, College Station, TX, USA). The intention-to-treat analysis was conducted by an independent statistician. For continuous data, the statistical analysis was performed using the Student *t*-test and median regression. Additionally, categorical data were compared using the chi-squared test and Fisher’s exact test, as appropriate. A value of *p* < 0.005 was considered statistically significant.

## 3. Results

Between March 2020 and February 2021, 68 women were recruited, consented, enrolled, and were randomly allocated into either the early transurethral catheter removal group (early removal group) or the conventional removal group, with 34 patients in each group. No participants in this cohort were excluded; therefore, outcome data were available for all 68 women (Figure 1).

**Figure 2:**
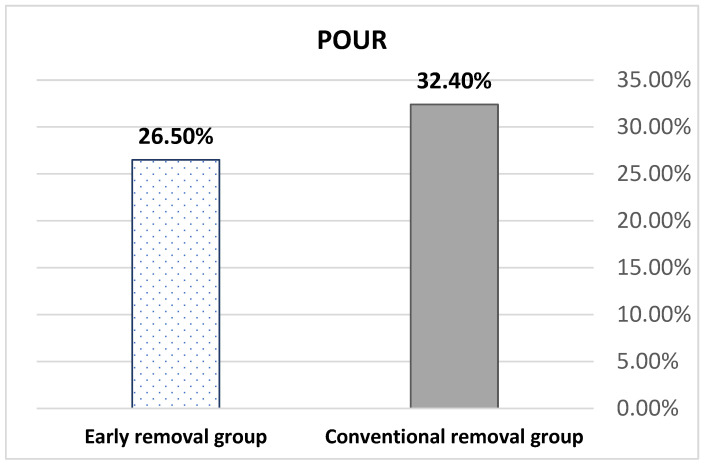
Incidence of postoperative urinary retention (POUR).

The average participant age was 66.7 ± 9.94 years. There were no statistically significant differences in baseline characteristics (Table 1). All patients underwent anterior colporrhaphy or colpocleisis. There were no differences in terms of intraoperative blood loss, operative time, anesthetic modalities, opioid use, or complications (Table 2). The primary outcomes illustrated that POUR occurred in 20 women (29.4%), of whom 11 (32.4%) were in the conventional group and 9 (26.5%) were in the early removal group (Figure 2). The POUR rate of the conventional group was 18.2% higher than that in the early removal group; however, this was not statistically significant (relative risk = 0.82; 95% CI: 0.39–1.72). The 20 women with POUR were discharged home with a retained Foley catheter, and all of them returned to spontaneous voiding after 3 days of follow-up.

There was no significant difference between groups in terms of the postoperative AB rate (14.7 vs. 0%, *p* = 0.053). No postoperative UTI developed in any patients. The early removal group had shorter hospital stays (1 day vs. 3 days, *p* < 0.001) and a 3.6 h earlier time to ambulation (*p* = 0.2), with no significant differences in postoperative patient satisfaction, as assessed by PGI-I scores (*p* = 0.58); most women reported that they were “much better” (Table 3). Even though some patients experienced POUR, they were still satisfied with the outcome (80% vs. 87.5% in patients with and without POUR, respectively; *p* = 0.465). Other postoperative complications after anterior compartment prolapse surgery were detected in the early removal group, namely, vaginal hematoma and blood transfusion. Two patients (5.9%) and 0 patients had vaginal hematoma in the early removal and the conventional groups, respectively (*p* = 0.49), and blood transfusion rates were 2.9% (1 patient) and 0 patients, respectively (*p* = 1.00). There were no adverse events caused by the early catheter removal protocol.

## 4. Discussion

In recent years, studies have aimed to establish the optimal time of catheterization to balance the rates of POUR and catheter-associated UTI. A recent systematic review by Xie et al. [9] revealed that the preferable timing of catheter removal is within 2 days postoperatively. However, there were variations among studies that enrolled a concomitant anti-incontinence operation and various statistical heterogeneities. Therefore, this timing might not be generalized. Additionally, most previous studies were not specific to anterior compartment prolapse-related surgery [11,12]. According to our results, the rates of overall, conventional, and early removal POUR were 29.4%, 32.4, and 26.5%, respectively. The conventional removal group had a higher POUR rate, which may be owing to the longer operative time; however, there was no significant difference in the rate of urinary retention after anterior compartment prolapse surgery between groups. In a randomized study among patients who underwent anterior compartment prolapse-related surgery with or without posterior colporrhaphy or vaginal hysterectomy, Hakvoort et al. [11] showed that 40% and 9% of patients needed recatheterization if the transurethral catheter was removed on the morning after surgery and the 5th postoperative day, respectively. In that study, POUR was defined as a PVR > 200 mL. In another study, Gourisankar [12] revealed a significantly higher number of retentions in the early removal group (1st postoperative day) than the conventional group (4th postoperative day), with 21.4% versus 8%; odds ratio 3.10. In that study, POUR was defined as a PVR > 150 mL, which was similar to the present study. However, only six patients were discharged with a retained Foley catheter. After 3 days of follow-up, all of these patients returned to spontaneous voiding. In a recent RCT study, Fernandez-Gonzalez [19] attended to postoperative protocol of anterior colporrhaphy; 24 vs. 48 h catheter removal after surgery. There were no significant differences in POUR rate (2.5 vs. 8.1%, respectively) (*p* = 0.346). The UTI and AB rates were also decreased. The result was roughly similar to ours. Nevertheless, these POUR rates are in contrast to the findings of our study owing to inconsistent catheter removal dates. Certain factors that might explain these variations in POUR rate are the difference in PVR cutoff values and the mean measurement when considering POUR. Also, our study included patients who were older at the time of surgery and had a greater number of preoperative advanced POP-Q stages compared with previous studies. Moreover, our study included all types of anterior compartment surgery, such as AC and colpocleisis, and details of concomitant surgery. Additionally, we adhered strictly to the protocol for the timing of catheter removal, 24 h postoperatively. Furthermore, the postoperative AB rate in our study did not show significant differences between groups (14.7 vs. 0%) (*p* = 0.053), and there were no postoperative UTIs. Patients in previous studies showed a lower risk of UTI development [9,15].

This study demonstrated that anterior compartment prolapse-related surgery performed as an overnight surgery appears to be safe and feasible. Additionally, we observed no re-hospitalization owing to POUR. The benefits were to reduce unnecessary, costly hospitalization and the bed occupancy rate, and to improve the patient turnover rate.

To our knowledge, no previous studies have assessed postoperative patient satisfaction. We used the PGI-I and found that the mean index response was “much better”, which was similar in both groups (*p* = 0.58). Moreover, patients who experienced POUR were still satisfied, with 80% and 87.5% in POUR and non-POUR patients, respectively; *p* = 0.465. This result may reflect good patient–doctor relationships, good surgical outcomes, preoperative counseling regarding the risk of developing POUR, and the use of a noninvasive tool to measure PVR.

The strengths of this study include the randomized controlled design with no dropouts. Because our institute is a university hospital, surgeons follow standardized surgical techniques under the supervision of well-trained FPMRS staff in every case. Nevertheless, this study also has some limitations. We did not have well-allocated intraoperative anesthetic modalities, and there was variation in the postoperative pain control protocol. However, these should not affect our results. However, we may not be able to determine which method is superior based on only the results of this trial, but our research will provide the data obtained from the trial, which could be utilized in future practical applications.

As for the clinical implications of our findings, POUR is a frequent consequence of transvaginal surgery. To prevent this complication, indwelling bladder catheterization is routinely used. In contrast, prolonged bladder catheterization may increase the likelihood of UTI development, prevent ambulation, prolong hospitalization, and also adversely affect postoperative well-being [9,10,15,20].

Future researchers should consider having a well-designed anesthetic and pain control protocol. Further research is needed into 1-day pelvic floor surgery with 24-h and 6-h transurethral catheter removal.

## 5. Conclusions

In conclusion, in patients undergoing anterior compartment prolapse-related surgery with or without concomitant surgery, patients with early catheter removal had POUR rates comparable to the conventional group and shorter lengths of hospital stay. Shorter hospitalization supports the current policy to reduce the bed occupancy rate. Therefore, early transurethral catheter removal is preferable following anterior prolapse surgery.

## Figures and Tables

**Table 1:** Baseline participant characteristics.

		Conventional Transurethral	Early Transurethral	*p* Value
		Catheter Removal (*n* = 34)	Catheter Removal (*n* = 34)	
Age (y), mean ± SD		65.7 ± 10.7	68.1 ± 9.4	0.33 ^a^
BMI (kg/m^2^), mean ± SD		25.0 ± 4.0	24.6 ± 3.6	0.71 ^a^
Vaginal parity, *n* (%)				
	0	1 (2.9%)	1 (2.9%)	1.00 ^c^
		3 (8.8%)	4 (11.8%)	
	≥2	30 (88.2%)	29 (85.2%)	
Menopause, *n* (%)		32 (94.1%)	34 (100%)	0.49 ^c^
Medical comorbidity, *n* (%)				
	Respiratory disease	4 (11.6%)	0	0.11 ^c^
	Metabolic disease	26 (76.5%)	28 (82.4%)	0.55 ^b^
	CVD	1 (2.9%)	6 (17.7%)	0.11 ^c^
Current drug use, *n* (%)				
	Alpha-blocker	1 (2.94%)	0	1.00 ^c^
	Beta-blocker	2 (5.9%)	4 (11.8%)	0.67 ^c^
Prior surgery, *n* (%)				
	Hysterectomy	5 (14.7%)	5 (14.7%)	1.00 ^b^
	Anti-incontinence surgery	1 (2.9%)	0	1.00 ^c^
	POP surgery			
	AC	1 (2.9%)	3 (8.8%)	0.61 ^c^
	PC	1 (2.9%)	1 (2.9%)	1.00 ^c^
Baseline anterior vaginal prolapse, *n* (%)				
	POP-Q stage 2	5 (14.7%)	9 (26.5%)	0.49 ^b^
	POP-Q stage 3	15 (44.1%)	13 (38.2%)	
	POP-Q stage 4	14 (41.2%)	12 (35.3%)	
Preoperative PVR (mL), median (range)		42.95 (0.45–149)	37.5 (0.97–121)	0.94 ^d^
Preoperative asymptomatic bacteriuria, *n* (%)		5 (14.7%)	6 (17.7%)	1.00 ^c^

^a^ *t*-test; ^b^ Pearson’s χ^2^; ^c^ Fisher’s exact test; ^d^ median regression. SD—standard deviation; BMI—body mass index; CVD—cardiovascular disease; POP-Q—pelvic organ prolapse quantification; AC—anterior colporrhaphy; PC—posterior colporrhaphy; PVR—postvoid residual.

**Table 2:** Operative data.

	Conventional Transurethral	Early Transurethral	*p* Value
	Catheter Removal (*n* = 34)	Catheter Removal (*n* = 34)	
Main operation, *n* (%)			0.45 ^c^
Anterior colporrhaphy	20 (58.8%)	24 (70.6%)	
Colpocleisis	14 (41.1%)	10 (29.4%)	
Total colpocleisis	13 (38.2%)	10 (29.4%)	
Lefort	1 (2.9%)	0	
Concomitant surgery, *n* (%)			
Vaginal hysterectomy	26 (76.5%)	25 (73.5%)	0.78 ^b^
McCall culdoplasty	1 (2.9%)	6 (17.6%)	0.11 ^c^
Sacrospinous ligament fixation	5 (14.7%)	2 (5.9%)	0.43 ^c^
Laparoscopic sacrocolpopexy	0	1 (2.9%)	1.00 ^c^
Manchester operation	0	1 (2.9%)	1.00 ^c^
Perineoplasty	9 (26.5%)	2 (5.9%)	0.02 ^b^
Posterior colporrhaphy	12 (35.3%)	11 (34.2%)	0.80 ^b^
Uterosacral ligament fixation	1 (3.13%)	0	1.00 ^c^
Intraoperative factors			
Operative time (min), mean ± SD	108.1 ± 37.1	90.6 ± 33.7	0.05 ^a^
Blood loss (mL), median (range)	50 (10–300)	50 (10–200)	1.00 ^d^
Intraoperative IV fluid (mL), median (range)	1002.9 ± 342.0	869.7 ± 379.3	0.13 ^d^
Intraoperative urine output (mL), median (range)	300 (0–900)	300 (50–900)	1.00 ^d^
Anesthetic modality, *n* (%)			
General anesthesia	9 (26.5%)	13 (38.2%)	0.05 ^c^
SB without intrathecal morphine	17 (50.0%)	20 (58.8%)	
SB with intrathecal morphine	8 (23.5%)	1 (2.9%)	
Opioid use (equianalgesic to IV morphine)			
Intraoperative use, median (range)	0 (0–10)	0 (0–10)	1.00 ^d^
Postoperative use, median (range)	6 (0–30)	4 (0–37.3)	0.61 ^d^
Complications, *n* (%)			
Vaginal hematoma	0	2 (5.9%)	0.49 ^c^
Blood transfusion	0	1 (2.9%)	1.00 ^c^

^a^ *t*-test; ^b^ Pearson’s χ^2^; ^c^ Fisher’s exact test; ^d^ median regression. SD—standard deviation; IV—intravenous; SB—spinal anesthesia.

**Table 3:** Postoperative outcomes.

	Conventional Transurethral	Early Transurethral	*p* Value
	Catheter Removal (*n* = 34)	Catheter Removal (*n* = 34)	
POUR, *n* (%)	11 (32.4%)	9 (26.5%)	0.60 ^a^
Time to ambulation (h), median (range)	28 (6–60)	24.4 (15.3–28)	0.20 ^c^
Time to spontaneous voiding (h), median (range)	1.5 (0.5–4.3)	2 (0.5–5.75)	0.06 ^c^
Length of hospitalization (d), median (range)	3 (3–4)	1 (1–5)	<0.001 ^d^
Patient satisfaction (PGI-I), median (range)	2 (1–4)	2 (1–3)	0.58 ^c^
Postoperative asymptomatic bacteriuria, *n* (%)	5 (14.7%)	0	0.05 ^b^

^a^ Pearson’s χ^2^; ^b^ Fisher’s exact test; ^c^ median regression; ^d^ Wilcoxon rank-sum test. PGI-I—Patient Global Impression Scale of Improvement; POUR—postoperative urinary retention.

## Data Availability

Data is available upon reasonable request from corresponding author (orawee.chi@mahidol.ac.th).

## Abbreviations

POP: pelvic organ prolapse; POP-Q: pelvic organ prolapse quantification system; AC: anterior colporrhaphy; POUR: postoperative urinary retention; UTI: urinary tract infection; PVR: postvoid residual volume; PGI-I: Patient Global Impression Scale of Improvement, patient’s satisfaction questionnaire after anterior prolapse surgery; AB: asymptomatic bacteriuria; FPMRS: female pelvic medicine and reconstructive surgery.
